# Pulmonary Thromboembolism Presenting with Recurrent Bradycardia and Hypotension

**Published:** 2017

**Authors:** Alireza Khosravi, Elham Andalib, Arsalan Khaledifar, Majid Hajizadeh, Majid Nejati, Mohaddeseh Behjati

**Affiliations:** 1 Interventional Cardiology Research Center, Cardiovascular Research Institute, Isfahan University of Medical Sciences, Isfahan, Iran; 2 Hypertension Research Center, Cardiovascular Research Institute, Isfahan University of Medical Sciences, Isfahan, Iran.; 3 Hajar Hospital, Shahrekord University of Medical Sciences, Shahrekord, Iran; 4 School of Medicine, Abadan University of Medical Sciences, Khouzestan, Iran; 5 Anatomical Sciences Research Center, Kashan University of Medical Sciences, Kashan, Iran; 6 Rajaie Cardiovascular, Medical and Research Center, Iran University of Medical Sciences, Tehran, Iran.

**Keywords:** Pulmonary thromboembolism, Bradycardia, Syncope

## Abstract

Recurrent short episodes of bradycardia and hypotension are rarely reported as clinical manifestations of pulmonary thromboembolism (PTE). We report a case of acute massive PTE presenting with dyspnea, burning chest pain with physical activity, and recurrent transient bradycardia and hypotension at rest. Echocardiography showed a left ventricular ejection fraction of 45% with global hypokinesia. Computed tomography angiography showed a large pulmonary thromboembolism. Lytic therapy improved the right ventricular function, and the pulmonary artery pressure decreased to 38 mmHg. Recurrent bradycardia and transient hypotension at rest with syncope on activity and recovery without treatment are not common and may suggest a vasovagal mechanism. Evaluation of patients with these clinical findings could enable early diagnosis and treatment of acute PTE, with decreased morbidity and mortality.

## INTRODUCTION

Acute pulmonary thromboembolism (PTE) is a common and potentially lethal condition in which early diagnosis and proper management could reduce the mortality rate (1). About half of all the cases are idiopathic and occur without antecedent trauma, surgery, immobilization, or a diagnosis of cancer (2). Unfortunately, the diagnosis is often missed because patients with PTE usually present with nonspecific signs and symptoms. If left untreated, about one-third of patients who survive will die from a subsequent embolic episode (3). Common signs and symptoms of pulmonary embolism include dyspnea, pleuritic chest pain, tachycardia, and tachypnea. Recurrent short episodes of bradycardia and hypotension are rarely reported as clinical manifestations of PTE (4). We describe a case of acute massive PTE presenting with dyspnea, burning chest pain with physical activity, and recurrent transient bradycardia and hypotension at rest.

## CASE SUMMARIES

A 61-year-old physically active man without a history of smoking or pulmonary disease presented with exertional chest heaviness and dyspnea while walking. The symptoms were relieved with a brief rest, and the patient could continue walking. His past medical history was significant only for seasonal allergy. His heart rate (HR) was 87 beats/min, with blood pressure (BP) 130/80 mmHg and respiratory rate (RR) 16/min. Cardiac examination was normal. The electrocardiogram (ECG) showed nonspecific ST-T changes. Coronary angiography revealed normal arteries. However, echocardiography showed a left ventricular (LV) ejection fraction of about 45% with global hypokinesia. During hospitalization, he repeatedly experienced symptoms of dyspnea, chest heaviness, bradycardia, and hypotension during defecation. He also experienced recurrent cold sweats with dyspneic episodes and hypotension.

These clinical symptoms continued during his hospitalization. During one episode, his HR was 38 beats/min, with BP 60/40 mmHg and RR 40/min. Hypotension was treated with 500 ml of intravenous normal saline. Seven hours after coronary angiography, the patient had a syncopal episode while walking in the hospital. He recovered normal sinus rhythm and blood pressure without treatment. After repeated episodes of bradycardia and hypotension, the physical examination demonstrated a right ventricular (RV) heave, elevated jugular venous pressure, a tricuspid regurgitation (TR) murmur at the left sternal border, and an S1Q3T3 ECG pattern with right bundle branch block. Repeat echocardiography showed RV enlargement with free-wall hypokinesia and systolic pulmonary artery pressure (PAP) of 60 mmHg. The serum D-dimer level was immediately measured using a Tina-quant D-Dimer kit and a 400 Cobas Integra analyzer (both from Roche- USA).

Lower extremity color Doppler ultrasound showed thrombosis in the right posterior tibial artery. Computed tomography (CT) angiography confirmed the presence of a large pulmonary thromboembolism. The patient was treated with streptokinase (250,000 U bolus and then 100,000 U/h), followed by heparin infusion (1,000 U/h for 5 days, in addition to warfarin). Repeat echocardiography showed improvement of RV function, and the PAP decreased to 38 mmHg. On the sixth hospital day, follow-up lower extremity color Doppler ultrasound showed normal results, without any thrombosis. The patient was discharged from the hospital on day 10 with daily warfarin. On follow-up, he was in normal sinus rhythm and appeared well.

**Figure 1.**
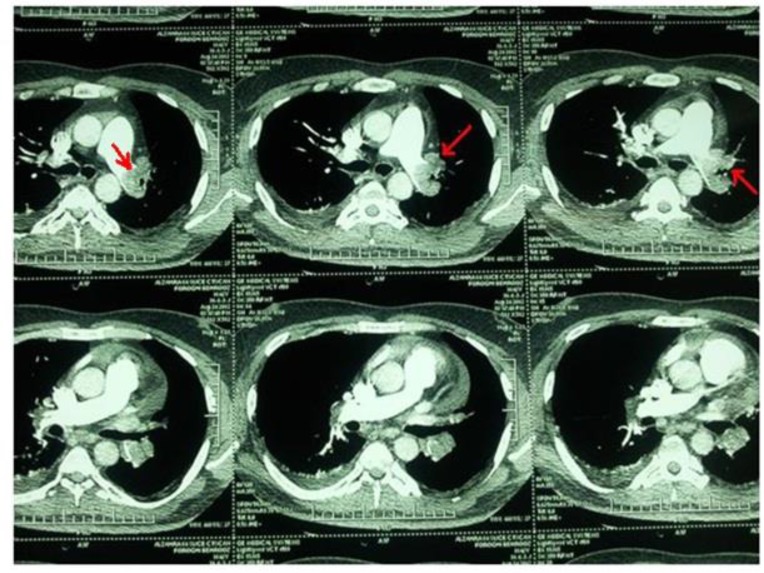
CT angiography confirmed the presence of large pulmonary thromboembolism

## DISCUSSION

PTE diagnosis based on clinical and laboratory findings is a medical challenge (2). Among the different clinical symptoms in patients with PTE, dyspnea, tachypnea, and tachycardia are most common. Although syncope is seen in 10% of patients with PTE (3), recurrent symptomatic bradycardia and hypotension are rarely reported. PTE is not rare, but it is often overlooked in the differential diagnosis of syncope.

Syncope in patients with PTE can be caused by several different mechanisms: acute RV failure due to massive PTE can occur after a prolonged duration of hypotension; bradycardia and complete atrioventricular block can occur in patients with a history of bundle branch block, as reported by Thoren et al. and Hubloue et al.; or vasovagal syncope can occur with an increase in heart rate and LV contractility (5, 6).

In the present case, recurrent bradycardia and syncope could be due to recurrent vasovagal reflex stimulation caused by repeated PTE episodes, and the syncopal attack may have been due to another major episode. Akinbibiye et al. reported a patient with second degree block and syncope who was diagnosed with PTE (7).

## CONCLUSION

Patients with acute and recurrent PTE have dyspnea, tachypnea, and tachycardia as the most common clinical presentations. Recurrent bradycardia and transient hypotension at rest with syncope on activity and recovery without treatment are not common and could suggest a vasovagal mechanism. Patients who present with the above unusual clinical findings should be evaluated for acute PTE, as early diagnosis and treatment can decrease morbidity and mortality.
